# Structured Tandem Repeats in Protein Interactions

**DOI:** 10.3390/ijms25052994

**Published:** 2024-03-05

**Authors:** Juan Mac Donagh, Abril Marchesini, Agostina Spiga, Maximiliano José Fallico, Paula Nazarena Arrías, Alexander Miguel Monzon, Aimilia-Christina Vagiona, Mariane Gonçalves-Kulik, Pablo Mier, Miguel A. Andrade-Navarro

**Affiliations:** 1 Science and Technology Department, National University of Quilmes, Bernal B1876, Argentina; macjuan17@gmail.com (J.M.D.); agostina.spiga@becarios.unq.edu.ar (A.S.); 2 National Scientific and Technical Research Council (CONICET), Buenos Aires C1033AAJ, Argentina; abrilmarchesini@biol.unlp.edu.ar; 3 Biotechnology and Molecular Biology Institute (IBBM, UNLP-CONICET), Faculty of Exact Sciences, University of La Plata, La Plata 1900, Argentina; 4 Laboratory of Bioactive Compound Research and Development, Faculty of Exact Sciences, University of La Plata, La Plata 1900, Argentina; maxifallico@gmail.com; 5 Department of Biomedical Sciences, University of Padova, Via U. Bassi 58/b, 35121 Padova, Italy; 6 Department of Information Engineering, University of Padova, Via Giovanni Gradenigo 6/B, 35131 Padova, Italy; alexander.monzon@unipd.it; 7 Institute of Organismic and Molecular Evolution, Faculty of Biology, Johannes Gutenberg University, Hans-Dieter-Hüsch-Weg 15, 55128 Mainz, Germany; avagiona@uni-mainz.de (A.-C.V.); magoncal@uni-mainz.de (M.G.-K.); munoz@uni-mainz.de (P.M.)

**Keywords:** tandem repeats, protein–protein interactions, protein structure, protein evolution, protein flexibility

## Abstract

Tandem repeats (TRs) in protein sequences are consecutive, highly similar sequence motifs. Some types of TRs fold into structural units that pack together in ensembles, forming either an (open) elongated domain or a (closed) propeller, where the last unit of the ensemble packs against the first one. Here, we examine TR proteins (TRPs) to see how their sequence, structure, and evolutionary properties favor them for a function as mediators of protein interactions. Our observations suggest that TRPs bind other proteins using large, structured surfaces like globular domains; in particular, open-structured TR ensembles are favored by flexible termini and the possibility to tightly coil against their targets. While, intuitively, open ensembles of TRs seem prone to evolve due to their potential to accommodate insertions and deletions of units, these evolutionary events are unexpectedly rare, suggesting that they are advantageous for the emergence of the ancestral sequence but are early fixed. We hypothesize that their flexibility makes it easier for further proteins to adapt to interact with them, which would explain their large number of protein interactions. We provide insight into the properties of open TR ensembles, which make them scaffolds for alternative protein complexes to organize genes, RNA and proteins.

## 1. Introduction

Tandem repeats (TRs) in protein sequences are approximate consecutive repeats, which can fold into units with similar folding packing together in a structural domain [1,2,3]. In 2012, Kajava categorized TRs that form structures according to their lengths into class I (length of 1–2 amino acids, forming crystalline structures), class II (length of 3–4 amino acids, fibrous proteins), and longer ones that can form open (class III) or closed (class IV) ensembles [3]. Open ensembles include solenoids, in which the array of TRs is elongated in one direction. Their units can be composed of a pure helical [4] or beta structure [5], or they can be mixed (e.g., Leucin Rich Repeats; LRRs [6]). Solenoids have a large surface and flexibility that allows them to interact with multiple proteins simultaneously as scaffold proteins [7,8,9]. As a result, they interact with many proteins for a variety of cellular functions in many species widely distributed across the tree of life [10,11,12]. 

Well-known examples are importin alpha (which controls the traffic of proteins into the nucleus and binds proteins with a nuclear localization signal using Armadillo repeats [13]), mammalian ribonuclease inhibitor (which tightly coils around ribonuclease with an ensemble of LRRs [14]) and Huntingtin (a large protein with several ensembles of HEAT repeats, mostly studied for its role in Huntington’s disease but with a large list of protein interactors, likely to play a role coordinating multiple cellular processes [15]).

Because each unit in ensembles of repeats has evolved to interact with its neighbours, ensembles of repeats can easily accommodate the insertion or deletion of a unit. This gives them an evolutionary advantage over globular domains because the ensemble of repeats can increase or decrease in size without the need for complex rearrangements of the folding of the domain, which would not be possible for a globular domain [16,17]. This is probably one of the reasons why TRs are so ubiquitous. Accordingly, an enrichment of proteins with conserved TRs in functions related to binding was observed [18]. Because TR proteins (TRPs) have a variety of types and particular properties, we wondered if the association of TRs to an interaction function could be related to general or specific properties of TRs giving them advantages for a function in protein interactions. To address this question, here we evaluate the properties of TRPs in relation to protein interactions.

## 2. Results

### 2.1. Comparison of the Properties of TRs and Other Protein Regions

We obtained the complete set of human proteins (a total of 20,600) and identified 11 types of structured TRs (Ankyrin, Armadillo, HAT, HEAT and three variants thereof, Kelch, LRR, PFTA, PFTB, RCC1, TPR and WD40) in these sequences using REP2 [19] (see [20] for details about these TR types). There are other methods for the detection of tandem repeats (e.g., TAPO [21], RepeatsDB-Lite [22], CE-Symm [23], and TRDistiller [24]), but for the purposes of this work, using a single method such as REP2, which detects and assigns TRs in protein sequences to a few well-defined categories of well-known structural repeats, is sufficient.

**Figure 1:**
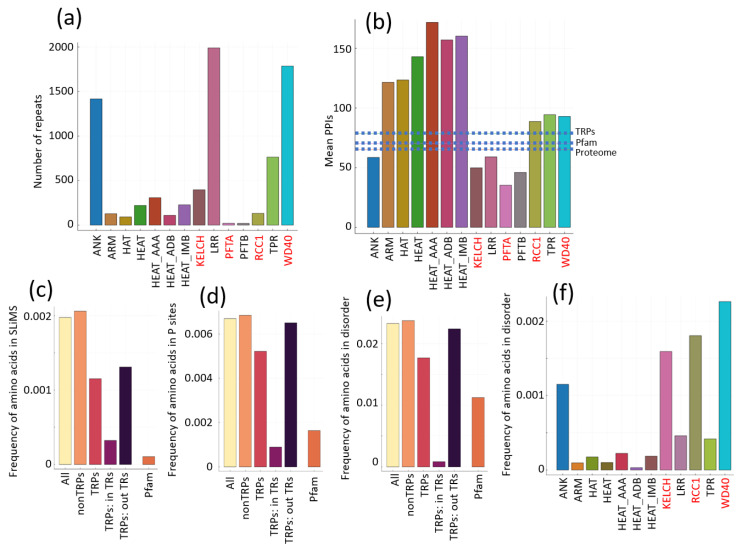
Properties of TRs by type and compared to other protein regions. (**a**) Number of repeat units identified. HEAT_AAA, HEAT_ADB and HEAT_IMB are three variants of HEAT repeats and can be redundant. TRs forming open and closed ensembles are indicated with black and red labels, respectively. (**b**) Average number of protein partners for TRPs. Horizontal dotted lines indicate the values for all human proteins (proteome), for proteins annotated with globular domains (Pfam), and for all TRPs. (**c**) Frequency of amino acids in SLiMs. Values are shown for: the complete human proteome (All), for proteins that do not contain TRs (non TRPs), for TRPs, for residues in TRs of TRPs (TRPs: in TRs), for residues outside TRs in TRPs (TRPs: out TRs), and in annotated globular domains (Pfam). (**d**) Frequency of amino acids in phosphorylation sites. (**e**) Frequency of amino acids in IDRs. (**f**) Disordered content by repeat type (PFTA and PFTB are not displayed because their numbers are too low). To prepare the plots (**c**–**f**), we obtained the coordinates of the features (SLiMs, phosphorylation sites, disorder regions) in all human sequences from the corresponding databases (see Methods for details) and then divided the number of residues within the given feature by the total of residues in the corresponding type of sequence.

We detected a total of 7749 repeat units in 1005 proteins (with some redundancy). WD40, LRR and Ankyrin were the most abundant (Figure 1a; Supplementary Table S1). 

Regarding the amount of protein interactions of proteins with these TRs (TRPs), we observed a large variability (Figure 1b). Open TRPs with ARM, HEAT and TPRs had more than 100 interactions on average, well above the average human protein (65, n = 20,596), proteins with annotated Pfam domains (70, n = 17,329) and TRP (79, n = 1005; Supplementary Table S2). However, open TRPs with ANK and LRR have below average numbers of protein–protein interactions (PPIs). Thus, in this respect, while TRPs have more interactions on average than the global human proteome, this is not specific to open or closed TRPs.

To identify properties of TRs relevant to protein interactions and how they compare to regions outside TRs and to globular domains, we defined different sequence ranges and computed the frequency of various annotations inside and outside TRs.

First, we studied the presence of Short Linear Motifs (SLiMs), which are sites for protein interactions [25,26], expecting to find an enrichment of those given the interacting character of TRs. We actually found the opposite result (Figure 1c). These motifs mediate interactions of a peptide with another protein [27]. We observed that globular domains have an even lower frequency of SLiMs than TRs (Figure 1c; see Methods for definition). 

For comparison, we also tested the frequency of phosphorylation sites, as these are often present in the interfaces of interacting proteins, resulting in phosphorylation-dependent PPIs [28,29]. These sites were also depleted of TRs, even more than in globular domains (Figure 1d). 

To further substantiate this observation, since SLiMs and phosphorylation sites are very frequent in disordered regions [30,31], we checked the content of disorder in TRPs. While both IDRs and TRs can have a function in PPIs, they differ in that TRs have a structure, and therefore IDRs impose stronger constraints on the proteins that have them. For example, proteins with long IDRs have a shorter half-life [32].

We observed that the structured TRs analyzed have even less disorder content than globular domains (Figure 1e). TRs forming closed ensembles (KELCH, RCC1 and WD40) had the largest frequency of disorder (Figure 1f). 

Together, these results suggest that TRs are involved in interactions using folded surfaces similarly to globular domains and less like disordered regions. In fact, regarding the properties analyzed, TRs are further from IDRs than globular domains.

We note that the regions of TRPs outside the TR ensemble have a similar disorder content and phosphorylation sites to the average protein but a lower content of SLiMs (not so low as Pfam domains; Figure 1c). Since SLiMs tend to accumulate in IDRs, this is an indication that the IDRs of TRPs are depleted of SLiMs, suggesting that they would be less involved in PPIs mediated via SLiMs. We take this result as an indication that the TRs in TPRs are mainly responsible for their PPI function to the detriment of their IDRs, which would fulfil a more passive function, such as providing flexible linkers.

### 2.2. Flexibility

While our results indicate that TRPs behave as folded proteins in their interaction properties, they are well known to have a certain degree of flexibility, particularly those that form solenoids [2,33]. We wondered if there could be different properties in terms of the flexibility of TRs in open and closed structures. Restricted mobility of central residues in elongated ensembles of TRs has already been observed and is hypothesized to play a role in their interacting properties [34]. To examine the flexibility of TR ensembles, we studied known structures of human proteins with open and closed ensembles of TRs. As a normalized proxy for flexibility, we used the pLDDT scores in their corresponding AlphaFold predictions [35]. 

**Figure 2:**
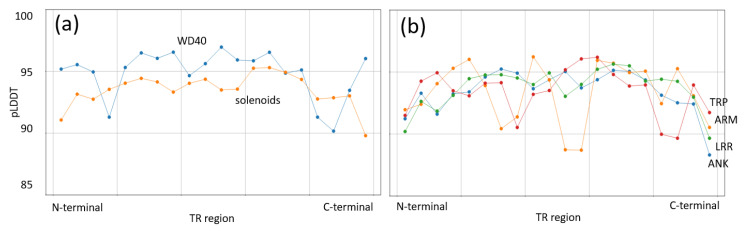
pLDDT scores of AlphaFold predictions along ensembles of TRs. (**a**) WD40 (closed ensemble) compared to an average of four open ensembles (shown in (**b**)). (**b**) Values for the four open ensembles. The x-axis indicates the relative position in the TR ensemble N- to C-terminal.

Considering the number of cases available, we were able to obtain enough data for one closed TR type (WD40 n = 31) and four open TR types (ANK n = 25, ARM n = 11, LRR n = 24, TRP n = 20). The average of pLDDT scores along the TR ensemble have values of around 93 with drops to 90 at the termini (Figure 2a). Individual open-ensemble TR types have some drops in the middle part, but the drops at the termini are found in all of them (Figure 2b). For comparison, the profile for the closed TR (WD40) has no such drops at the termini and displays slightly higher values (Figure 2a). 

These results suggest that TRs that form open ensembles might be more flexible than closed ones, particularly at the termini. This could result in their ability to grasp proteins in their center and then coil the termini around. Disordered regions are often found at the termini of TR ensembles, which could contribute to this effect [11]. 

**Figure 3:**
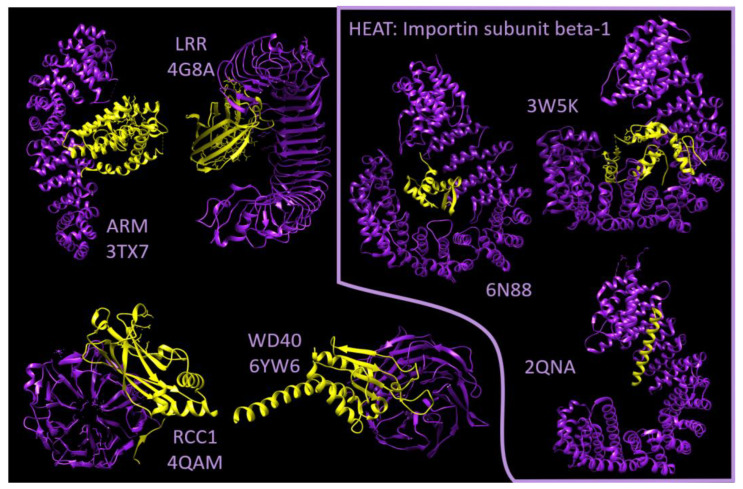
Flexibility of interacting TRPs. Structures of protein complexes with TRPs by repeat type. Elongated: ARM repeats in human catenin beta-1 binding NR5A2 (PDB:3TX7); LRR repeats in Toll-like receptor 4 binding LY96 (PDB:4G8A); HEAT repeats in importin subunit beta-1 shown in the same orientation, forming three complexes with histone H1.0 (PDB:6N88), Zinc finger protein SNAI1 (PDB:3W5K) and the IBB domain of Snurportin-1 (PDB:2QNA). Cyclic: RCC1 in RPGR repeats binding the interacting domain of RPGRIP1 (PDB:4QAM) and KELCH repeats in ARPC1B binding ARPC4 (PDB:6YW6). TRP in purple and bound protein in yellow.

We illustrate this with examples of TRPs in complex with another protein (as annotated in RepeatsDB [36]; Figure 3). The elongated ensembles of repeats (ARM, LRR, HEAT) can coil around their bound proteins, whereas closed ensembles (RCC1, WD40) form a more compact domain that behaves more like a globular domain. We illustrate the flexibility of elongated TRPs showing structures of the HEAT repeat domain of importin subunit beta-1 (C-terminal at the top of the image) bound to three different proteins (histone H1.0, SNAI1 and Snurportin-1; Figure 3, right side): both the binding location and the shape of the TRP change in each interaction. This property is very specific to elongated TRPs.

### 2.3. Length Variability

The evolutionary adaptability of TRPs for changes in the number of TR units is yet another property of open ensembles of TRs that distinguishes them from close TRs and makes them appealing molecules for protein interactions [7,37].

Changes in the number of units of ensembles of TRs have been observed by examination of orthologs from species at long divergence times (see e.g., for the mineralocorticoid receptor in human and fish [38]). With the sequencing of many complete genomes for species, strains and populations, it has also become possible to assess the fast evolution of proteins by comparing sequences from closely related organisms; one recent example in relation to sequence repeats is the examination of the variation of short TRs across different populations of the plant *Arabidopsis thaliana* [39]. 

To add examples specific to the structured TRs studied here and to illustrate the evolutionary variation between closely related species, we used the complete proteomes of six species of *Plasmodium*. The study of TRs in these species is particularly interesting because of their involvement in malaria. Clarifying features that could impart *Plasmodium* with evolutionary variability may contribute to an understanding of its pathogenicity in terms of adaptability to the host. In *Plasmodium*, short tandem repeats are variable [40], and here we examine whether this is the case for structured TRs.

We grouped their complete proteomes by sequence similarity (see Methods for details), predicted TRs in their sequences and then found cases where the number of predicted TRs was different between orthologs.

**Figure 4:**
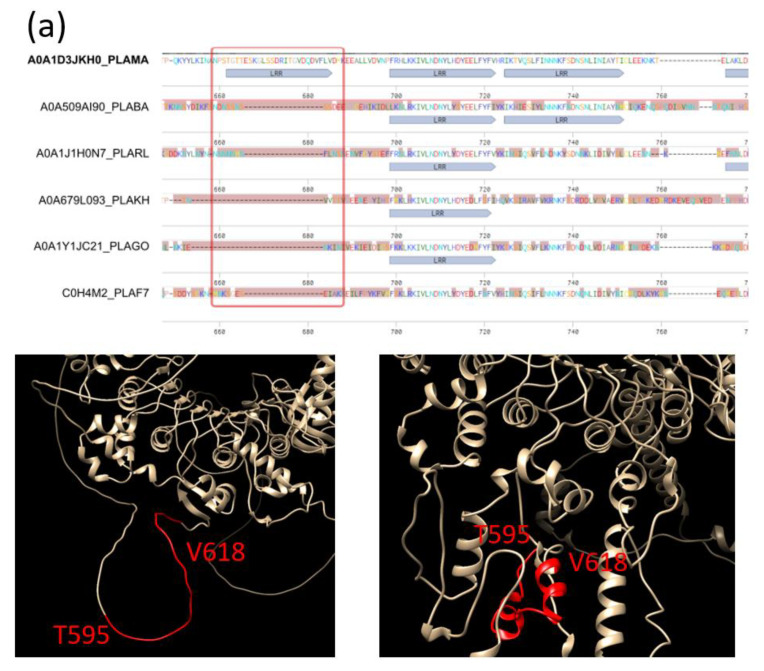

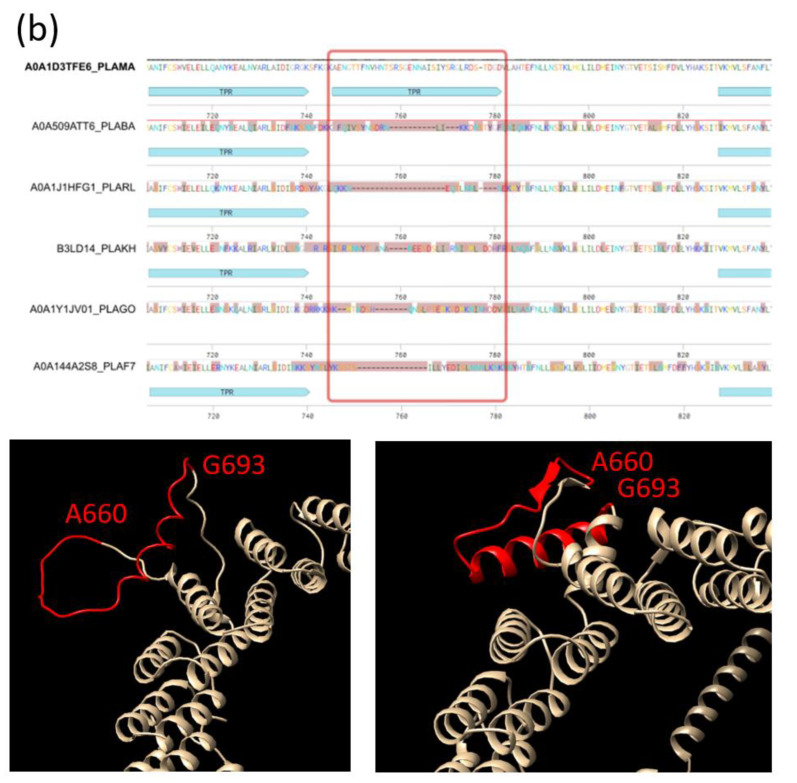
Cases of fast evolution in *Plasmodium* species. (**a**) Gain of an LRR unit in A0A1D3JKH0_PLAMA positions 595–618. (**b**) Gain of a TPR unit in A0A1D3TFE6_PLAMA positions 660–693 (colored in red in the structure). Sequence identifiers from UniProtKB. In order, species are: *Plasmodium berghei*, *Plasmodium relictum*, *Plasmodium malariae*, *Plasmodium knowlesi*, *Plasmodium gonderi* and *Plasmodium falciparum*. No alternative spliced isoforms for A0A1D3JKH0_PLAMA or A0A1D3TFE6_PLAMA are given in UniProt (February 2024). The structures shown are models from AlphaFold [41] and Robetta [42] (left and right, respectively).

We illustrate this fast evolution of TRs with two examples (Figure 4). The two cases regard open ensemble types (LRR and TPR, respectively), which is not surprising as it is easier to add or remove units to open ensembles than to closed ones, which have a more restricted folding [7]. Regarding their taxonomic distribution, LRRs are particularly predominant in plants, and TPRs are particularly predominant in Archaea and Bacteria, while in *Plasmodium falciparum* WD40 repeats (which form a closed ensemble) are actually more frequent [19].

In both cases, we have the insertion of a new unit in the ortholog of the *P. malariae* protein, and the region seems to be surrounded by disorder (Figure 4). The AlphaFold structure predictions [41] for the *P. malariae* sequences are available in UniProt (versions AF-A0A1D3JKH0-F1 and AF-A0A1D3TFE6-F1, respectively). None of them predict the insertion as a TR unit, and therefore our sequence-based prediction could be incorrect. The AlphaFold prediction for our extra predicted LRR in A0A1D3JKH0_PLAMA positions 595–618 does not find this LRR, but it has difficulties in predicting other repeats (Figure 4a; bottom left). However, the prediction for the A0A1D3TFE6_PLAMA sequence with the extra TPR in positions 660–693 suggests some helical structure (Figure 4b; bottom left). 

We also obtained predictions using the Robetta structure prediction server (using RoseTTAFold [42]). These predictions contain less disorder. Interestingly, in both cases, unlike AlphaFold, Robetta predicts a compact unit, formed by two secondary structure elements packed in an anti-parallel way against each other, with the start and the end of the unit in close proximity in 3D (Figure 4a,b; bottom right). However, the predicted secondary structures (alpha-alpha and beta-alpha, respectively) are not those of the repeats in the ensemble (LRR and TPR, respectively).

These two examples have common features that suggest that insertions in open ensembles of TRs could contain extra TRs. Structural predictions by AlphaFold do not confirm them, but alternative structural predictions by Robetta suggest that the insertions might contain units with the size and anti-parallel packing of the TRs in the ensemble. Therefore, while these extra TRs could be false positives from the sequence detection method, it is not possible to completely rule out that disordered insertions in TRs could be a seed for the evolution of new TRs. 

## 3. Discussion

**Figure 5:**
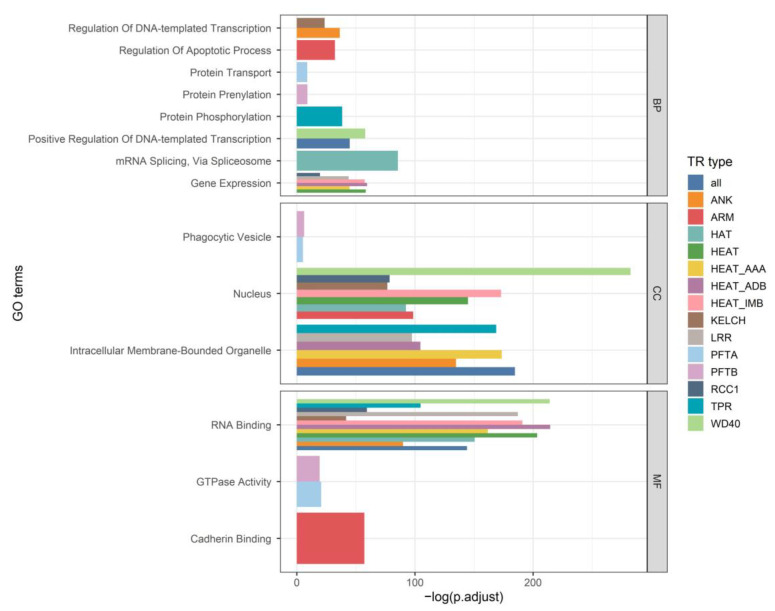
Functional enrichment of TRP interactors. **Top**: Biological Process. **Middle**: Cellular Component. **Bottom**: Molecular Function. Gene Ontology (GO) enrichment analysis was carried out for a set of TRP interactors (All) and then separately for the interactors of each TRP type (see Methods for details). Enriched GO Biological Process (BP), Molecular Function (MF) and Cellular Component (CC) terms with the lowest adjusted *p*-value were kept.

The capacity for TRPs to interact with other proteins favors their participation in functions that require the formation of protein complexes. The examination of the functional enrichment of proteins that interact with human TRPs (see Methods for details) indicates that they often operate in processes in the nucleus involving RNA binding (Figure 5). This is in agreement with the observation that most TRPs bind RNA, but through disordered regions and not directly with structural TRs [43]. As a result, functions in the regulation of transcription, expression and splicing are found. Protein regulation functions are associated with ARM (regulation of apoptosis) and TPR (phosphorylation).

The lack of evolutionary variation in the number of TR units we exemplified in *Plasmodium* has also been observed at long evolutionary distances in eukaryotes [18] and in plants [44]. While unit number variation might be advantageous for the establishment of the ancestral interaction, further evolution of TRPs seems to be rare. This is likely related to the mode of interaction of TRPs, which we found shares some aspects with that of globular domains. While it might be simple to modify the TRP side of a protein–protein interaction, the globular non-TRP interacting partner is not free to change, and thus the interacting interface is frozen, meaning that none of the partners will evolve. If both interacting partners are TRPs (or TRPs interact with an IDR), we could expect the evolutionary constraints to be much lower. Fast evolutionary rates of TRPs have been observed in giant viruses (e.g., ANK in the *Acanthamoeba polyphaga mimivirus*; [45]); we would hypothesize that the function of these proteins is to interact with other TRPs or IDRs, or maybe not with any protein.

Our observations provide a two-sided view of TRs. On the one hand, they provide modes of interaction by complementarity to large-structured surfaces (similar to globular domains) and by not using motifs and sites of phosphorylation (as disordered regions do). This results in a lack of evolutionary capacity. While inherent evolutionary plasticity exists, this is restricted to the generation of an ancestral surface for interaction that remains fixed.

On the other hand, elongated TRs have flexibility at long range, which allows them to interact with many partners and adopt different shapes depending on the interacting partners. Although the TRP is evolutionarily fixed, TRP flexibility can be exploited by novel partners adapting to interact with existing TRPs. The advantage here is that even if the length of the TRP cannot change, the conformational variability offers more possibilities for those new interactors that can bend the TRPs to their interacting needs. This would explain the fact that TRPs in general have more interactors than non-TRPs. Our results and interpretations support a bilateral view of open TRs, explaining their success and the multiplicity of TR types that have emerged in evolution.

## 4. Materials and Methods

A total of 20,600 human proteins were obtained from the human reference proteome from the UniProtKb release 2022_04 [46]. We identified 11 types of TRs with structural information (Ankyrin, Armadillo, HAT, HEAT and three variants thereof, Kelch, LRR, PFTA, PFTB, RCC1, TPR and WD40) in these sequences using REP2 [19]. A total of 7749 repeat units were detected in 1005 proteins.

Protein interactions between human proteins were obtained from the HIPPIE database [47]. SLiMs were evaluated using the ELM database [48], and phosphorylation sites were evaluated using the Phospho ELM database [49]. Globular domains were obtained from the InterPro annotations of Pfam domains in sequences [50]. Disordered regions were obtained using a consensus of predictors as defined in MobiDB [51]. Known structures of human TRPs and annotated domains of TRs were obtained from RepeatsDB [36]. Multiple sequence alignments were produced using Clustal Omega [52].

For the study of TRs in *Plasmodium* species, we obtained the complete reference proteome (not considering isoforms) of the following six species from UniProtKB release 2022_04: *Plasmodium berghei* (strain Anka), *Plasmodium falciparum* (isolate 3D7), *Plasmodium gonderi* (ATCC 30045), *Plasmodium knowlesi* (strain H), *Plasmodium malariae*, and *Plasmodium relictum* (SGS1). A total of 37,588 sequences were retrieved. They were grouped in groups of orthologs using OrthoFinder [53], and TRs were predicted in them using REP2 [19].

Interactors with human TRPs were obtained from the HIPPIE database [47]. Functional enrichment of protein sets was computed using Enricher [54]. 

## Data Availability

Data are contained within the article and Supplementary Materials.

## Supplementary Materials

The following supporting information can be downloaded at: https://www.mdpi.com/article/10.3390/ijms25052994/s1.
